# Decompressive Craniectomy in the Management of Low Glasgow Coma Score Patients With Extradural Hematoma: A Review of Literature and Guidelines

**DOI:** 10.7759/cureus.33790

**Published:** 2023-01-15

**Authors:** Yuganshu T Bisen, Paresh Korde, Onkar Dighe, Sandeep Iratwar, Ghrunanshu Bisen

**Affiliations:** 1 Department of Neurosurgery, Jawaharlal Nehru Medical College, Datta Meghe Institute of Medical Science, Wardha, IND; 2 Department of Neurosurgery, Varun Arjun Medical College, Shahjahanpur, IND

**Keywords:** craniotomy, traumatic brain injury, glasgow coma score, guidelines, neurosurgery, decompressive craniectomy, extradural hematoma

## Abstract

An extradural hematoma (EDH), also known as an epidural hematoma, is a collection of blood between the inner skull table and the dura mater. It is restricted by the coronal, lambdoid, and sagittal sutures, as these are dural insertions. EDH most frequently occurs in 10- to 40-year-old patients. EDH is uncommon after age 60, as dura matter adheres firmly to the inner skull table. EDH is more common among men as compared to women. EDH most commonly occurs in the temporo-frontal regions and can also be seen in the parieto-occipital, parasagittal regions, and middle and posterior fossae. An EDH contributes approximately 2% of total head injuries and 15% of total fatal head injuries. In EDH, patients typically have a persistent, severe headache, and also, following a few hours of injury, they gradually lose consciousness. The primary bleeding vessels for EDH are the middle meningeal artery, middle meningeal vein, and torn dural venous sinuses. EDH is one of the many consequences of severe traumatic brain injuries that might lead to death. EDH is potentially a lethal condition that requires immediate intervention as, if left untreated, it can lead to growing transtentorial herniation, diminished consciousness, dilated pupils, and other neurological problems. Non-contrast computed tomography (NCCT) imaging is the gold standard of investigation for diagnosing EDH. For patients with surgical indications, early craniotomy and evacuation of acute extradural hematoma (AEDH) is the gold standard procedure and is predicted to have significant clinical results. Nevertheless, there is an ongoing debate regarding the best surgical operations for AEDH. Neurosurgeons must choose between a decompressive craniectomy (DC) or a craniotomy to manage EDH, especially in patients with low Glasgow coma scores, to have a better prognosis and clinical results. This is a consultant-based review article in which we have tried to contemplate various pieces of available literature. Here, the objective is to hypothesize DC as the primary surgical management for massive hematoma, which usually presents as a low Glasgow coma score. This is because DC was found to be beneficial in clinical practice.

## Introduction and background

Extradural hematoma (EDH) is a neurosurgical emergency that can result in an abrupt rise in intracranial pressure (ICP) and a local mass effect, necessitating immediate intervention to decrease morbidity and mortality [1]. The BTF has prepared an educational guideline on the surgical indications for acute extradural hematoma (AEDH). Patients can be managed conservatively with serial non-contrast computed tomography (NCCT) scans only if the patient has a Glasgow Coma Score (GCS) of more than eight, no focal impairment, and a hematoma less than 30 cm3 in volume, less than 15 mm thick, and less than 5 mm of midline shift under neurological supervision in neurosurgical facilities. According to the Brain Trauma Foundation (BTF), independent of the patient's GCS, a hematoma of more than 30 cm3 in volume should be surgically removed [2]. Anisocoric patients with AEDH in a coma with a Glasgow Coma Score less than 9 and a volume of more than 30 cm^3^, more than 15 mm thick, and more than 5 mm of midline shift are strongly advised for surgery, as immediate surgical intervention and intensive care are being found to be linked with improved clinical outcomes and a decreased fatality rate in patients with AEDH in a poor clinical state. Uncontrollable intracranial hypertension makes surgery tricky despite recent advancements in treating AEDH [3]. Many adverse complications, including cerebral ischemia and decreased cerebral blood flow, have been linked to an elevated ICP. The authors of various research suggest that early and prompt control of ICP and cerebral blood flow is crucial for enhancing patient outcomes and is linked to better results [4]. EDH is more concerning than subdural hematoma due to the fact that EDH crosses the falx cerebri and displaces the dural venous sinuses, leading to dysfunction of the flax cerebri and further worsening the prognosis [5-7].

After craniotomy (CO) with the evacuation of hematoma, patients usually experience clinical deterioration due to postoperative complications like cerebral infarction and brain edema, especially if the patient had preoperative herniation [8]. And for the management of those complications, decompressive craniectomy (DC) is employed. The authors of this research suggest that instead of performing CO, patients should primarily be managed with DC. Primary management of EDH using DC will decrease the hospital stay and the chances of developing secondary complications, leading to better clinical outcomes. Also, several clinical studies have found that a DC may lower morbidity and death in critically ill patients with severe head injuries. However, only a small percentage of surgeons decide to manage patients with DC in patients of AEDH. This is due to a lack of high-quality evidence addressing the use of DC in AEDH, which causes considerable variations in practices among countries, institutions, and even neurosurgeons in the same hospital. Another critical issue is identifying populations that may or may not benefit from DC, and further research will be needed to address this issue [9].

## Review

Methodology

**Figure 1:**
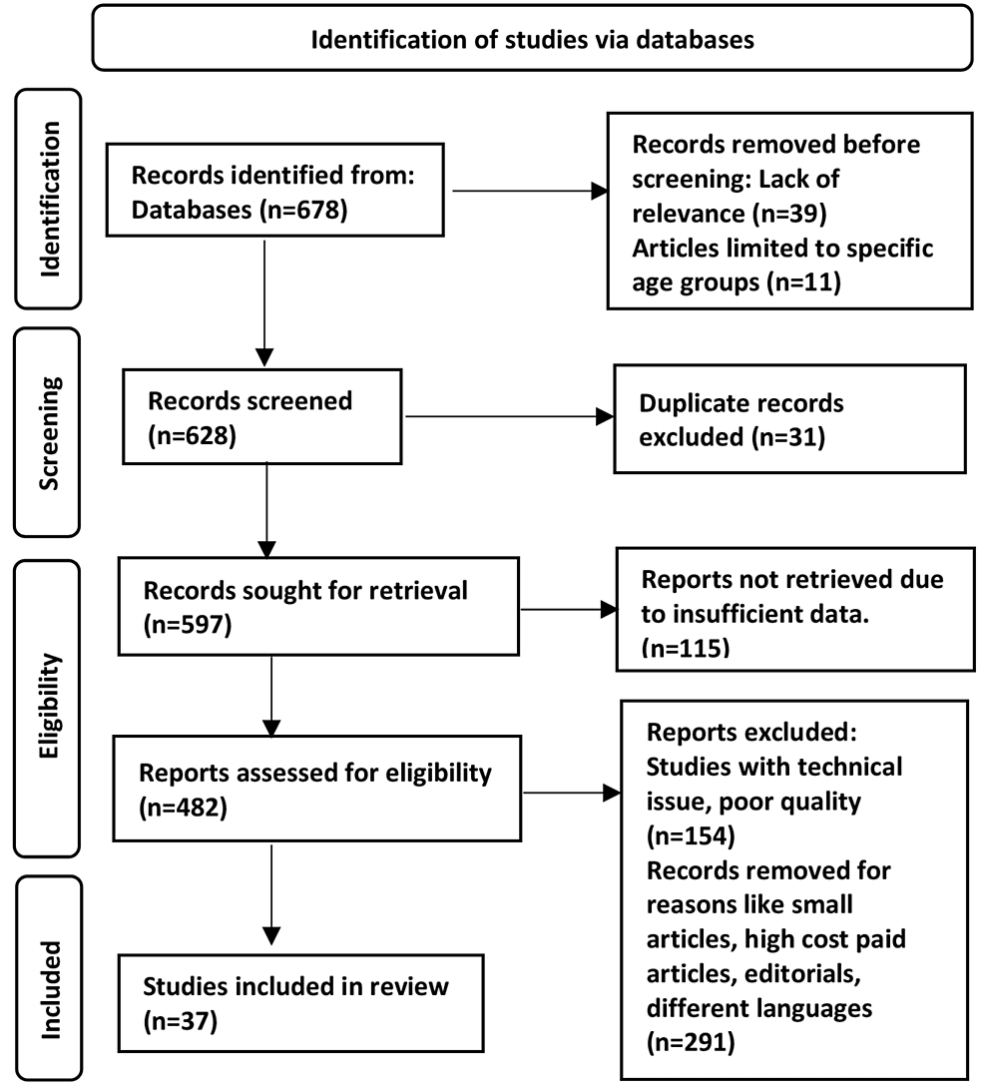
PRISMA model for search strategy. PRISMA: Preferred Reporting Items for Systemic Reviews and Meta-Analyses.

The following keywords were used in the Embase, Scopus, Cochrane, Google Scholar, and advanced PubMed searches: head injury or traumatic brain injury (TBI), extradural or epidural, hematoma or hemorrhage, and decompressive craniectomy, excluding the keywords spinal, intraparenchymal, subarachnoid, and subdural. The search yielded 678 articles, of which 37 research publications were selected for research. The methodology of the Preferred Reporting Items for Systemic Reviews and Meta-Analyses (PRISMA) method is shown in Figure 1.

Diagnosing EDH

Diagnosing EDH plays a critical role in managing the patient (i.e., if the patient has to be managed conservatively or surgically). Patients present in casualty with a concern of intracranial injury, the most common cause being road traffic accidents. Other causes of EDH include sports injuries, physical assault, falls from heights, and firearm-related injuries [10]. TBI either causes bending or fracture of the skull, which leads to stripping of the dura from the skull, which in turn leads to bleeding from the torn vessels in pockets formed between the inner skull table and dura matter [11,12]. Clinical features in patients with EDH include clouding of consciousness; the patient can be first conscious, then unconscious, or initially unconscious, then recover. Other clinical features include focal neurological deficits, seizures, visual field defects, aphasia, weakness, numbness, headache, vomiting, hyperreflexia, positive Babinski sign, bradycardia (due to elevated ICP), and associated extracranial injuries like zygomatic, nasal, mandible, and maxillary fractures [13].

All suspected TBI patients should be evaluated in compliance with advanced trauma life support (ATLS) recommendations. Routine blood tests, such as a complete blood count, urine examination, c-reactive protein, clotting profile, liver function tests, and renal profile, are indicated as a part of primary investigations. Plain brain radiographs are not usually helpful. An NCCT head can quickly rule out an EDH and identify associated intracranial lesions. AEDH is described as biconvex lens-shaped hyperdense lesions on NCCT scans [14]. The temporo-parieto-frontal convexity is the most common site where lesions are easily detected on axial sections. Additional coronal views may be necessary to identify the rare hematoma over the vertex. Large EDH may show a significant mass effect with evidence of herniation. Another critical element is the scheduling of the second NCCT scan. As reported in the recently published article, repeat NCCT imaging is helpful, as 23% of patients undergo EDH enlargement. This repeat imaging is required to ascertain if the patient is still qualified for conservative management. Enlargement of the EDH begins eight hours after injury and is completed within 36 hours [15]. Various differential diagnoses for EDH include post-traumatic and subdural hematomas.

Management of EDH

Depending upon the GCS, the volume of the hematoma, the thickness of the EDH, the location of the EDH, the mid-line shift, and the repeat imaging (NCCT), the patient can be managed either conservatively or surgically. Urgent neurosurgical advice is necessary upon patient stabilization and confirmation of the EDH. The BTF's latest suggestions state that not all EDH need surgery. Regardless of other variables, surgical management for EDH of more than 30 cm3 (volume) is advised. Candidates for conservative care include hematomas less than 30 cm3 in volume with low thickness and limited midline shift, a GCS of eight and above, and no localized neurological impairments [16-18]. For a patient with significant EDH, evidence of neurological deterioration, and transtentorial herniation, the patient should be transferred to the operation theatre for immediate removal of hematoma by exploratory burr hole under NCCT [19]. Table 1 summarises the BTF guidelines.

**Table 1:** Factors affecting the management of EDH. mm: millimeters; cm: centimeters; EDH: extradural hematoma [8].

Factors	Conservative management	Operative management
Midline shift on computed tomography	Less than 5 mm of midline shift	More than 5 mm of midline shift
The volume of hematoma	Less than 30 cm^3^ can be considered for conservative management	More than 30 cm^3 ^should be surgically evacuated
The thickness of the hematoma	Less than 15 mm in thickness	More than 15 mm thickness
Clinical findings	Glasgow coma score of eight and above can be managed considering the above factors	Glasgow coma score less than nine with anisocoria

Conservative Management

One may debate the need for surgical decompression in patients who are discovered to have EDH and are conscious with no neurological deficit, as these patients are most likely eligible for conservative management. Refrain from a conservative approach if the hematoma is large [20,21]. Conservative management should be undertaken in specialized neurocritical care units. Also, before contemplating additional therapy options, the patient with a suspected EDH should be stabilized using the airway, breathing, circulation, disability, and exposure method (ABCDE). Preventative antibiotics may be used, particularly in an open skull fracture, to reduce the likelihood of intracranial infections. Patients with AEDH are more likely to experience seizures. Hence, anticonvulsant medications like lamotrigine, levetiracetam, and carbamazepine may be started to avoid seizures in these patients. Hypertonic saline is indicated to reduce raised ICP by osmotic effect. Barbiturates may also be used to lower ICP and cerebral hypoxia. In the case of pediatric patients, posterior and temporal EDH are suitable for conservative management, except in the case of brainstem compression [22].

Operative Management

A CO is considered the gold standard procedure and is usually performed to manage AEDH operatively. It helps to drain the hematoma and treat the cause of the bleeding, eventually decreasing ICP. The operation of a choice craniotomy with hematoma removal involves taking out a portion of the patient's skull, removing the blood clot, preventing significant bleeding, and replacing the skull bone with microscopic screws [9]. Drilling burr holes in the head is another surgical technique to reduce raised ICP, as the pressure that a blood clot puts on the brain is relieved by draining the blood through burr holes [23]. Another surgical approach is DC, often defined as the temporary removal of a significant portion of the skull. DC has long been a tool in the arsenal of neurosurgeons for managing the increased ICP due to TBI. There are two types of DC. Primary DC is a procedure conducted soon after head trauma in which an intracranial mass lesion is evacuated. Unlike in CO, the bone flap is not replaced here. Later in the patient's course of the illness, secondary DC involves the removal of the bone flap to treat a refractory ICP. DC aids in overcoming the skull's rigid and non-compliant characteristics. In spite of that, the DC has generated debate throughout the past century [24-26]. Disagreements still continue to exist regarding the technical components of the surgery, time, and patient selection. There has even been discussion regarding whether this treatment should ever be undertaken for EDH [27].

The Operative Procedure of CO

Under general anesthesia, an incision is made directly above the EDH. An adequate portion of the skull is removed (craniotomy). The blood clot is located and released. The diathermy is used to halt bleeding at any prominent place. After that, titanium plates and skin staples are used to replace and fix the bone [28].

The Operative Procedure of DC

The patient should be positioned on the operating table such that the part of the skull with the underlying hematoma is on top. The skin and the bone flap are created such that they surround the hematoma. One inch anterior to the external auditory meatus, a vertical incision measuring 7 cm long and extending to the zygomatic process is made. The incision should not be continued past the zygomatic process to prevent cutting of the facial nerve branches that innervate the frontalis and orbicularis oculi muscles. The temporalis muscle and fascia fibers are severed and pulled back using a self-retaining instrument. The burr hole is made broader and more angular. After the CO is completed down to the bottom of the temporal fossa, the hematoma is suctioned out. The EDH should be quickly decompressed, rapidly reducing the ICP and improving outcomes. Hence, the hole should be created as soon as possible. The single burr hole can be converted into a CO flap using a trephine. It could be crucial to coagulate the middle meningeal artery here or at the foramen spinosum if it runs immediately above the fracture site. In other cases, there may be no apparent artery to allow the blood to clot; the bleeding may begin directly from the foramen spinosum. In some circumstances, bone wax is injected into the foramen spinosum to halt the bleeding. Applying bone wax to the fracture site will also stop the bleeding. A tiny incision in the dura should be made to check for an acute subdural hematoma once the clot has been removed and sufficient hemostasis has been achieved. The dura must be wrapped around the edge of the CO. One dural stitch can be applied near the center of the exposed dura; it will pass through responsive holes close to the center of the craniotomy flap. Further, Table 2 compares the differences between DC and CO.

**Table 2:** Comparison of decompressive craniectomy and craniotomy. Source [28].

Decompressive craniectomy	Craniotomy and evacuation of hematoma
The skull bone is removed in decompressive craniectomy to prevent brain tissue compression. Here skull bone is replaced usually six months after surgery.	The temporary removal of skull bone is known as craniotomy. Here before the surgery is finished, the bone is replaced.
Complications include brain edema, hematoma expansion, cerebral herniation, epilepsy, hydrocephalus, brain abscess, and sepsis.	Complications include secondary brain edema, infections, bleeding, seizures, permanent neurological damage, and death.
Need two different operations.	It can be done at one time.
Costly.	Cost-effective.
Fewer chances of secondary injuries like brain edema.	More cases of secondary brain injuries.

Postoperative Management

Postoperative management of patients after CO or DC should be done in the specialized neurocritical care unit, as patients can have postoperative deterioration like hematoma, cerebral infarction, postoperative seizures, acute hydrocephalus, pneumocephalus, cerebral edema, and postoperative headache. To prevent these complications, patients are started on steroids like dexamethasone, antiepileptic drugs like lamotrigine, proton pump inhibitors like pantoprazole, prophylactic antibiotics like ceftriaxone, and non-steroidal anti-inflammatory medications like paracetamol. And to check the prognosis, laboratory investigations like a complete blood count, a renal profile, and arterial blood gases are done. Postoperative NCCT is also advised [22].

Controversies about the use of DC in EDH

The following are controversial studies on the use of DC after an EDH that offer benefits and drawbacks. According to prior research, DC only provides good neurological outcomes for patients with an initial GCS higher than five [29]. The few examinations that addressed the function of DC in post-TBI came to the following main conclusions: (1) when intensive medical care failed, decompression had to be done, usually on young patients with a GCS of at least seven and no obvious ICP signs of irreversible brain damage; (2) timing, age, and perioperative ICP might significantly affect the outcome of surgery; and (3) the focus of the treatment had to be on preserving a stable ICP. Other investigations have shown that patients with DC and a low initial GCS of eight had poor clinical outcomes (extremely high morbidity and death rates) [30,31]. As reported by Otani et al., individuals with a GCS of three and unresponsive bilateral pupils had a mortality rate of 100% after DC [13]. Severe TBI injuries presenting with a GCS of three or four have been regarded in neurosurgery and critical care as challenging for patients. Due to the reported low clinical outcomes and the low possibility of functional restoration, DC has been avoided [3].

Benefits of DC for EDH

Clinical studies suggest that a DC may decrease morbidity and mortality in seriously ill individuals with significant head injuries [32,33]. Typically, DC results in a substantial increase in the middle cerebral artery pulsatility index values and a dramatic reduction in the mean cerebral blood flow velocity, indicating a decrease in cerebrovascular resistance. According to the surgical outcome, DC for an AEDH in 263 patients has improved brain tissue oxygenation and decreased ICP significantly. According to recent clinical research, an external decompression and extensive duroplasty might lower the ICP and boost cerebral tissue oxygenation [34,35]. According to the data from the case series, surgical therapy that includes a sufficiently wide temporo-parieto-frontal DC, hematoma evacuation, and appropriate post-operative management may benefit patients with serious TBI and a GCS of three or four [36]. According to research by Albanese et al., DC allowed 10 out of 40 patients with refractory intracranial hypertension who were at a very high risk of brain death to achieve social rehabilitation after one year [7]. Much evidence from clinical trials suggests that early neuroprotective DC is not superior to medical management for mild to moderate ICP, but in the case of severe raised ICP (which clinically presents as a low GCS) and in the case of refractory ICP, it leads to a substantial reduction in mortality and morbidity [26]. In a case study, when ICP was greater than 30 mmHg for more than 15 minutes, a DC was performed on each of the 16 patients. At six months, three of them had died (19%), two had a severe disability (12%), four had a moderate disability (25%), and seven had made a good recovery (44%) [37]. Further, Table 3 summarises all beneficial and controversial studies on the use of DC in EDH.

**Table 3:** Studies on the use of DC in patients with EDH. GCS: Glasglow coma score; DC: decompressive craniectomy; TBI: traumatic brain injury; AEDH: acute extradural hematoma; ICP: intracranial pressure; EDH: extradural hematoma. Source: Original.

Author	Year	Study design	Study region	Result
Albanèse et al. [7]	2003	Retrospective cohort study	Marseilles, France	DC allowed 25% of patients in 40 with refractory intracranial hypertension at a very high risk of brain death to achieve social rehabilitation after one year.
Rossini et al. [25]	2019	Review article	Rozzano, Italy	Early neuroprotective DC is not superior to medical management for mild to moderate ICP, but in the case of severe ICP (which clinically presents as a low GCS) and in a refractory ICP leads to a substantial reduction in mortality and morbidity.
				The few studies that addressed the role of DC in post-traumatic diffuse brain injury came to the following main conclusions: (1) When intensive medical care failed, decompression had to be done, usually on young patients with a GCS of at least seven and no obvious ICP signs of irreversible brain damage (2) timing, age, and postoperative ICP, Might significantly affect the outcome of surgery and (3) The focus of the therapy had to be on preserving a stable ICP. Other investigations have shown that patients with DC and low initial GCS eight had poor clinical outcomes (extremely high morbidity and death rates).
Grille et al. [37]	2015	Retrospective study	Montevideo, Uruguay	When ICP was greater than 30 mmHg for more than 15 minutes, a DC was performed on each of the 16 patients. At six months, three of them had died (19%), two had a severe disability (12%), four had a moderate disability (25%), and seven had made a good recovery (44%).
Afif [35]	2019	Retrospective study	France	Surgical therapy that includes a sufficiently wide parietal-tempo-frontal DC, hematoma evacuation, and appropriate postoperative management may benefit patients with serious TBI and a GCS of three or four.
Stiefel et al. [34]	2004	Case-control study	Philadelphia, Pennsylvania	An external decompression and extensive duroplasty might lower the ICP and boost cerebral tissue oxygenation.
Ban et al. [29]	2010	Retrospective study	Rozzano, Italy	DC only provides good neurological outcomes for patients having an initial GCS higher than five.
Otani et al. [13]	2006	Comparative Study	Toronto, Canada	Individuals with three GCS and unresponsive bilateral pupils had a mortality rate of 100 percent.
Tuncer et al. [3]	1997	Review article	Antalya, Turkey	Severe TBI injury presenting with a GCS of three or four has been regarded in neurosurgery and critical care as challenging for patients. DC surgery has thus been avoided due to the reported poor results and a poor likelihood of functional restoration.

## Conclusions

Like any other consequence of TBI (intraparenchymal haemorrhage, subdural haematoma), EDH should be dealt with extreme caution. While managing the extradural hematoma, surgeons should make rational decisions considering severity, pre-operative characteristics, long-term outcomes, and complications, as both surgeries have advantages and disadvantages. It is debatable whether using DC in a patient with an EDH is beneficial compared to craniotomy and evacuation of haematomas, as patients who underwent CO achieved long-term benefits. Despite that, evidence suggests early and vigorous use of DC improves clinical outcomes and decreases the chances of developing secondary injuries. The authors of this article indicate that DC can potentially be the primary surgical management for extradural hematoma. Still, in-depth research will be required to warrant the use, as insufficient literature is available.
